# Long non-coding RNA breast cancer-associated transcript 54 sponges microRNA-1269b to suppress the proliferation of hemangioma-derived endothelial cells

**DOI:** 10.1080/21655979.2022.2027064

**Published:** 2022-02-24

**Authors:** Zhonjun Lv, Ke Yang, Ya Wang

**Affiliations:** Department of Vascular Surgery, Nanyang Central Hospital, Nanyang, Henan Province, China

**Keywords:** BRCAT54, miR-1269b, infantile hemangioma, proliferation

## Abstract

Long non-coding RNA (lncRNA) breast cancer-associated transcript 54 (BRCAT54) and microRNA-1269b (miR-1269b) are two critical ncRNAs in cancer biology, while their roles in hemangioma are unknown. Our preliminary sequencing data revealed their altered expression in hemangioma and predicted they could interact with each other. This study was therefore carried out to investigate the roles of BRCAT54 and miR-1269b in hemangioma, with a focus on their interaction. In this study, hemangioma samples donated by 20 infantile hemangioma patients at proliferating-phase and 20 infantile hemangioma patients at involuting-phase were used. The expression of BRCAT54 and miR-1269b in hemangioma samples, as well as hemangioma-derived endothelial cells (HDECs) and human umbilical vein endothelial cells (HUVECs) were detected by RT-qPCR. IntaRNA 2.0 was applied to predict the interaction between BRCAT54 and miR-1269b, which was then confirmed by RNA-RNA pulldown assay. Accumulation of BRCAT54 in the subcellular location of HDECs was detected by subcellular fractionation assay. The role of BRCAT54 and miR-1269b in cell proliferation has been explored by the BrdU assay. Compared to proliferating-phase tissues, involuting-phase tissues exhibited decreased expression levels of BRCAT54 and increased expression levels of miR-1269b. HDECs had decreased expression levels of BRCAT54 and increased expression levels of miR-1269b compared to that of HUVECs. In HDECs, BRCAT54, which was detected in both nuclear and cytoplasm fractions, directly interacted with miR-1269b. BRCAT54 and miR-1269b did not affect the expression of each other, while BRCAT54 suppressed the role of miR-1269b in enhancing the proliferation of HDECs. BRCAT54 may sponge miR-1269b to suppress the proliferation of HDECs.

## Introduction

As the most common type of tumor originates from the soft tissues in childhood, infantile hemangioma (IH) is also called hemangiomas of infancy [1]. IHs are caused by abnormally increased proliferation of endothelial cells, which is commonly observed in about 1 out of 10 children younger than 12 months old [2]. IHs have the ability to involute after proliferation [3–5]. Therefore, in the past, primary care providers usually assumed patients with IHs will recover without intervention [5]. Unfortunately, without proper treatment, some patients with IHs will develop complications, which may cause chronic pain, or even permanent disfigurement in extreme cases. Therefore, timely treatment is still needed [6]. Patients with IHs are usually treated with medicines, such as beta blockers [7]. However, serious neurological disorders may still develop after active treatment [8]. Therefore, novel therapies are of great importance.

The development of treatment approaches targeting IHs is limited due to the unclear molecular mechanisms [9,10]. Molecular targeted therapy is an emerging novel approach to treat human diseases. Some molecular pathways, such as the NF-κB signaling, have been characterized as potential targets to treat IHs [11]. However, molecular targeted therapy is still under research. The development of IHs involves altered expression of non-coding RNAs (ncRNAs), such as long ncRNAs (lncRNAs) and microRNAs (miRNAs) [12,13]. Some of these ncRNAs exert critical functions in IHs and therefore could be targeted to treat IHs. For instance, lncRNA CASC2 suppresses cell proliferation in IHs [14], while lncRNA OIP5-AS1 promotes cell IH cell proliferation [15]. Previous studies have characterized lncRNA breast cancer-associated transcript 54 (BRCAT54) and miR-1269b as two critical players in cancer biology [16–18], while their roles in hemangioma are unknown. Our preliminary sequencing data revealed their altered expression in hemangioma and predicted that they could interact with each other. Therefore, we speculated that BRCAT54 and miR-1269b may interact with each other to participate in hemangioma. The interaction between lncRNAs and miRNAs in IHs has rarely been studied. Increased elucidation of the molecular mechanisms of hemangioma may boost the development of novel approaches to treat this disease. The present study was then carried out to investigate the role of BRCAT54 and miR-1269b in hemangioma and the crosstalk between them.

## Materials and methods

### Patient samples

Hemangioma samples were donated by 20 IH patients at proliferating-phase (15 females and 5 males 5.6 ± 1.3 months old) and 20 IH patients at involuting-phase (15 females and 5 males, 5.7 ± 1.2 months old) who received surgical resection at Nanyang Central Hospital in Henan Province from May 2018 to August 2020. This study was approved by the Ethics Committee of this hospital. Patients’ parents signed the informed consent.

### Cells and transient transfection

Hemangioma-derived endothelial cells (HDECs) and human umbilical vein endothelial cells (HUVECs) were bought from the Cell Bank of Chinese Academy Sciences (Shanghai, China) and were cultivated in DMEM (Gibco) containing fetal bovine serum (10%) and penicillin–streptomycin (1%). Cell culture was carried out in an incubator with 5% CO_2_ and with temperature and humidity set to 37 ^º^C and 95%, respectively.

To overexpress BRCAT54 and miR-1269b, cells were transfected with miR-1269b mimic and/or pcDNA 3.1- BRCAT54 vector through transient transfections achieved with Neon Electroporation Transfection (Thermo Fisher Scientific). In each transfection, 10^6^ cells were transfected with 30 mM vector and/or 80 mM miRNA. The confirmation of overexpression (compared to NC mimic or empty vector transfection group) was performed every 24 h until the end of *in vitro* cell experiments.

### RNA isolation and sample processing

The MagNA Pure LC RNA Isolation Kit (Roche) was used to isolate total RNAs. Briefly, 10^7^ cultivated cells or 0.05 g tissue samples (ground in liquid nitrogen) were incubated with 10 volumes of lysis buffer containing Proteinase K. Magnetic glass particles were used to bind RNA, while DNase I digestion was performed to remove genomic DNA. RNA samples were subjected to Bioanalyzer analysis to determine RNA concentration and integrity. RNA concentrations higher than 1,000 ng/μl and RIN values higher than 9 were achieved in all samples.

### Reverse transcriptions (RTs)-qPCR

The synthesis of complementary DNA (cDNA) samples was performed with 2,000 ng total RNA sample through RTs using the qScript cDNA Synthesis Kit (Quantabio). All cDNA samples were tested by amplifying actin genes prior to subsequent assays. The expression of BRCAT54 and miR-1269b was measured by qPCR with 18S rRNA and U6 as the internal control, respectively. The 2^−ΔΔCT^ method [19] was applied to normalize Ct values to their corresponding internal controls. Primer sequences were as follows: 5’-GATATCCCAGAAGGTTATGC-3’ (forward) and 5’-AGGCTCCACCTCCAAACACC-3’ (reverse) for BRCAT54; 5’-CTACCACATCCAAGGAAGCA-3’ (forward) and 5’-TTTTTCGTCACTACCTCCCCG-3’ for 18S rRNA; 5’-CGGCAGCACATATACTAAAAT-3’ (forward) and 5’-GCTTCACGAATTTGCGTGTC-3’ (reverse) for U6; 5’-CTGGATGAGCCATGCTAC-3’ (forward) and poly (T) (reverse) for miR-1269b.

### Biotinylated RNAs for RNA-RNA pull-down assay

A T7 promoter vector expression BRCAT54 or NC RNA was used to prepare *in vitro* transcripts of both BRCAT54 and NC RNA. All *in vitro* transcriptions were performed using the HiScribe™ T7 High Yield RNA Synthesis Kit (NEB). The 3’-end of both RNA were biotinylated using the Biotin 3’ End DNA Labeling Kit (Life Technologies). Cells were then transfected with these two biotinylated RNAs (Bio-NC and Bio-BRCAT54) through the methods mentioned above. Cells were harvested 6 h later, followed by cell lysis on ice using lysis buffer. Centrifugations were performed on lysates, and the supernatants were collected and incubated with M-280 streptavidin magnetic beads (Sigma) coated with yeast tRNA and BSA (RNase-free). After that, beads were collected and washed with ice-cold PBS. Next, Trizol was used to purify the pulldown samples, followed by RT-qPCR to determine the expression of miR-1269b. Data normalizations were performed using the method mentioned above.

### Analysis of BRCAT54 expression in subcellular locations

The preparation of both cytoplasm and nuclear fractions from cells was performed using the cell fractionation kit (Cat #9038, Cell Signaling Technology). This kit separates cells into cytoplasmic, membrane/organelle, and nuclear/cytoskeletal fractions, while only cytoplasm and nuclear fractions were used in this study. The separation of the cytoplasm fraction was performed through a simple centrifugation at 800 g for 15 min on cell lysates. Nuclear fraction, which was the cell pellets after centrifugation, was collected for further nuclear lysis. Both fractions were used for RNA isolation through the methods mentioned above. After the synthesis of cDNA samples, PCR was performed to amplify BRCAT54. The PCR products were subjected to 1.5% agarose gel separations, followed by EB staining and MyECL image analysis.

### BrdU cell proliferation assay

DNA synthesis, which can be reflected by the incorporation of BrdU, was determined to reflect cell proliferation. Transfected cells were harvested at 48 h post-transfection. Cell culture was performed in a fresh medium for another 24 h, and then BrdU was added to reach a final concentration of 10 μM. Incubation with BrdU was performed for 2 h. After that the medium was removed, and cells were collected to incubate with peroxidase-coupled anti-BrdU antibody (Sigma–Aldrich). After incubation for 1 h, the cells were further incubated with a peroxidase substrate. Cell proliferation analysis was performed after 30 min of incubation by determining OD values at 450 nm.

### Statistics analysis

SPSS 17.0 statistical software (SPSS) was used for the analyses of all datasets. Student’s t-test was used to compare two groups, while multiple independent groups were compared by ANOVA Tukey’s test. The differences were statistically significant when *p* value < 0.05.

## Results

### The expression of BRCAT54 and miR-1269b in hemangioma samples, HDECs and HUVECs

**Figure 1.**
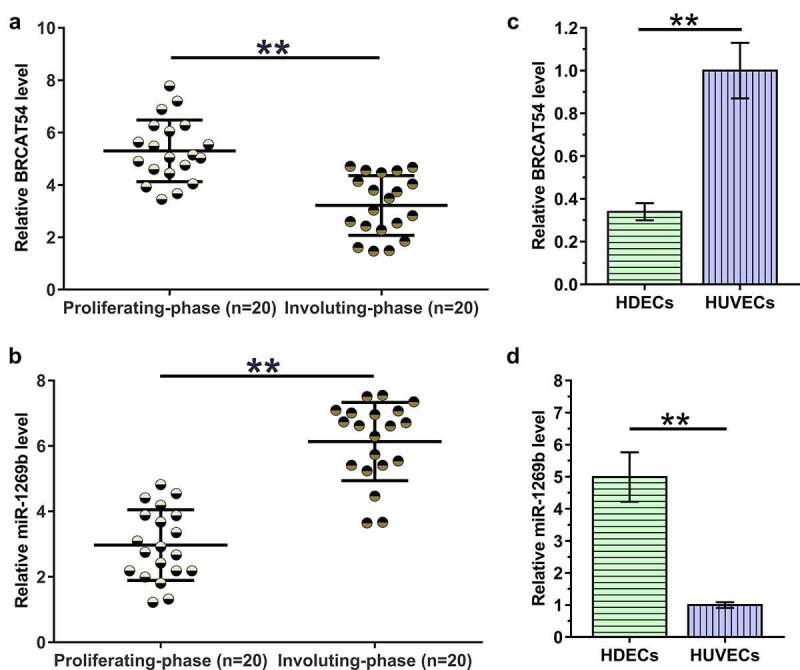
Analysis of BRCAT54 and miR-1269b expression in hemangioma samples, HDECs and HUVECs.

To analyze the differential expression of BRCAT54 and miR-1269b in IH, hemangioma samples from 20 IH patients at proliferating-phase and 20 IH patients at involuting-phase were subjected to RNA isolation and RT-qPCR. Compared to proliferating-phase tissues, involuting-phase tissues exhibited decreased expression levels of BRCAT54 (Figure 1a, *p* < 0.01) and increased expression levels of miR-1269b (Figure 1b, *p* < 0.01). The expression of BRCAT54 and miR-1269b in HDECs and HUVECs were also detected. The results showed decreased expression levels of BRCAT54 (Figure 1c, *p* < 0.01), and increased expression levels of miR-1269b (Figure 1d, *p* < 0.01) in HDECs compared to that in HUVECs. Therefore, BRCAT54 and miR-1269b may participate in the involuting-phase of IH.

Hemangioma samples from 20 IH patients at proliferating-phase and 20 IH patients at involuting-phase were subjected to RNA isolation and RT-qPCR to analyze the expression of BRCAT54 (a) and miR-1269b (b). The expression of BRCAT54 (c) and miR-1269b (d) in HDECs and HUVECs were also analyzed through the same method. **, *p* < 0.05.

### The subcellular location of BRCAT54 in HDECs and HUVECs, and its interaction with miR-1269b

**Figure 2.**
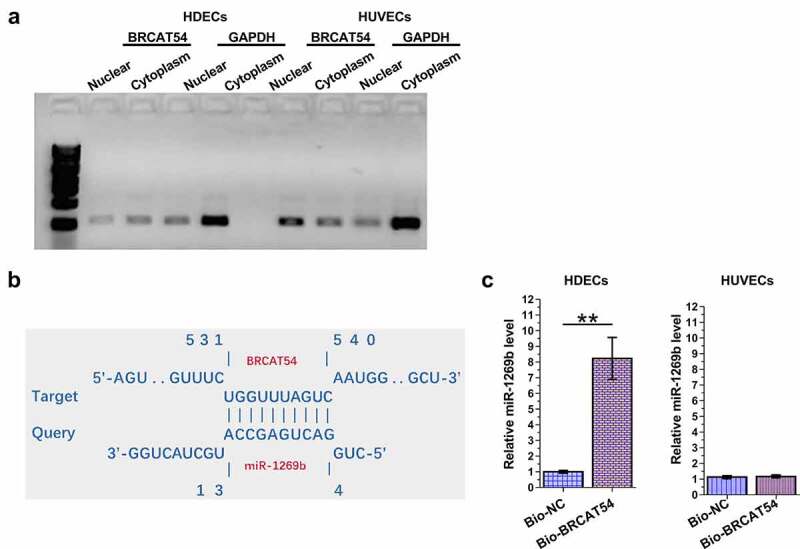
Analysis of the subcellular location of BRCAT54 in HDECs and HUVECs, and its interaction with miR-1269b.

The subcellular location determines the function. The subcellular location of BRCAT54 in HDECs and HUVECs was evaluated by subcellular fractionation assay. The data revealed that BRCAT54 can be detected in both cytoplasm and nuclear samples of both HDECs and HUVECs (Figure 2a). The online program IntaRNA 2.0 has been applied to predict the direct interaction between BRCAT54 and miR-1269b. It was predicted that BRCAT54 and miR-1269b could form multiple-base pairs (Figure 2b). RNA-RNA pulldown assay was performed to detect the direct interaction between BRCAT54 and miR-1269b, compared to Bio-NC group, Bio-BRCAT54 group showed significantly increased expression levels of miR-1269b in HDECs (*p* < 0.01), but not in HUVECs (Figure 2c). Therefore, BRCAT54 and miR-1269b may directly interact with each other in HDECs.

Subcellular location of BRCAT54 in HDECs and HUVECs was analyzed with subcellular fractionation assay (a). IntaRNA 2.0 online program was applied to predict the direct interaction between BRCAT54 and miR-1269b (b). RNA-RNA pulldown assay was performed to analyze the direct interaction between BRCAT54 and miR-1269b (c). **, *p* < 0.01.

### The role of BRCAT54 and miR-1269b in regulating the expression of each other

**Figure 3.**
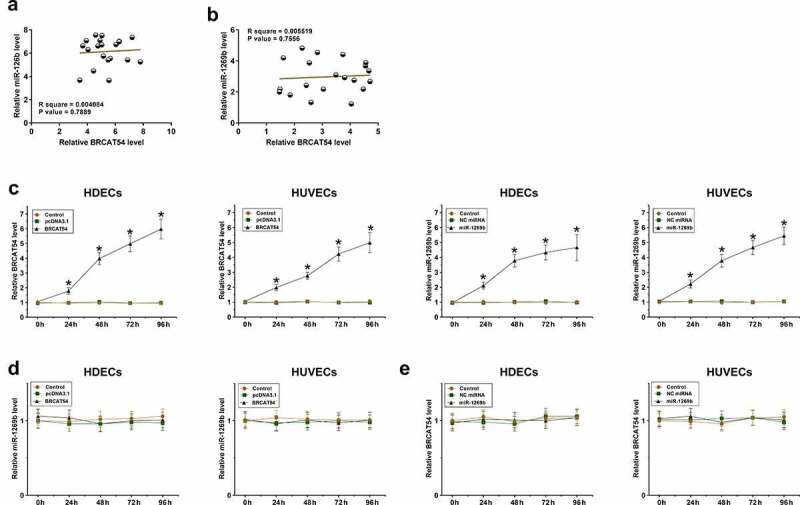
Analysis of the role of BRCAT54 and miR-1269b in regulating the expression of each other.

Correlations indicate possible interactions. The correlations between BRCAT54 and miR-1269b across both proliferating-phase and involuting-phase hemangioma samples were analyzed with Pearson’ correlation coefficient. It was observed that BRCAT54 and miR-1269b were not significantly correlated with each other across proliferating-phase (Figure 3a) or involuting-phase (Figure 3b) hemangioma samples. BRCAT54 and miR-1269b were overexpressed in HDECs and HUVECs, and the overexpression was confirmed every 24 h until 96 h (Figure 3c, *p* < 0.05). BRCAT54 did not affect the expression of miR-1269b in both cell types (Figure 3d) and miR-1269b showed no role in regulating the expression of BRCAT54 (Figure 3e). Therefore, BRCAT54 was unlikely to be a target of miR-1269b.

Correlations between BRCAT54 and miR-1269b across proliferating-phase (a) and involuting-phase (b) hemangioma samples were analyzed with Pearson’ correlation coefficient. BRCAT54 and miR-1269b were overexpressed in HDECs and HUVECs, and their overexpression was confirmed every 24 h until 96 h (c). The role of BRCAT54 in regulating the expression of miR-1269b (d) and the role of miR-1269b in regulating the expression of BRCAT54 (e) were analyzed with RT-PCR. *, *p* < 0.05.

### The role of BRCAT54 and miR-1269b in mediating the proliferation of both HDECs and HUVECs

**Figure 4.**
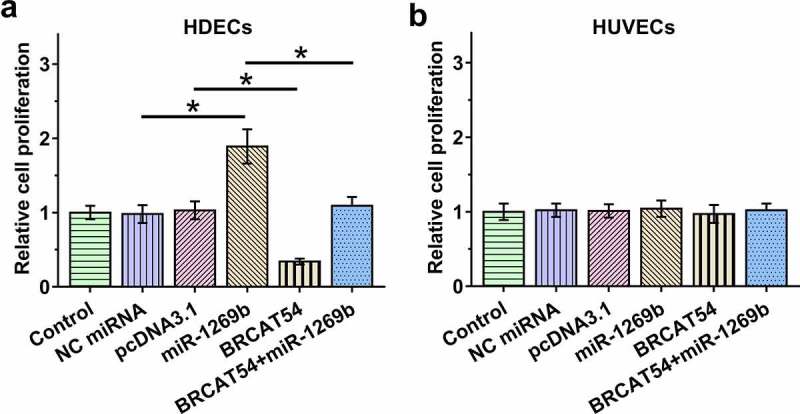
The role of BRCAT54 and miR-1269b in mediating the proliferation of both HDECs and HUVECs.

Cell proliferation determines the progression of IH. A BrdU assay was performed to explore the role of BRCAT54 and miR-1269b in regulating the proliferation of both HDECs and HUVECs. BRCAT54 inhibited the proliferation of HDECs, and it also suppressed the role of miR-1269b in enhancing the proliferation of HDECs (Figure 4a, *p* < 0.05). In contrast, BRCAT54 and miR-1269b showed no role in mediating the proliferation of HUVECs (Figure 4b). Therefore, BRCAT54 may regulate the proliferation of HDECs through miR-1269b.

A BrdU assay was performed to explore the role of BRCAT54 and miR-1269b in regulating the proliferation of both HDECs (a) and HUVECs (b). *, *p* < 0.05.

## Discussion

The present study explores the involvement of BRCAT54 and miR-1269b and the direct interaction between them in IHs. Our results show that the expression of BRCAT54 and miR-1269b were altered in IHs. BRCAT54 may sponge miR-1269b to regulate the proliferation of HDECs.

BRCAT54 is a recently characterized lncRNA in non-small cell lung cancer [16]. It was observed that BRCAT54 was downregulated in lung cancer and it regulates the expression of genes involved in the JAK-STAT and calcium pathways by binding to RPS9, thereby suppressing tumorigenesis [16]. However, the involvement of BRCAT54 in other types of cancer is unclear. Our results revealed decreased expression levels of BRCAT54 in hemangioma-derived HDECs compared to that in normal HUVECs. IHs have three development stages, including proliferation, plateau, and involution [20]. Our study also reported decreased expression levels of BRCAT54 in involution stage compared to that in the proliferation stage. Therefore, BRCAT54 is involved in IHs. In addition, the inhibitory effects of BRCAT54 on the proliferation of hemangioma-derived HDECs were observed. Therefore, BRCAT54 may serve as a target to treat IHs by suppressing cell proliferation.

MiR-1269b has been demonstrated to be an oncogenic miRNA in both liver cancer and lung cancer [17,18]. MiR-1269b is highly upregulated in these two types of malignancy, and it suppresses liver cancer growth and metastasis, and reduces the sensitivity of lung cancer cells to cisplatin by regulating the PTEN/PI3K/AKT signaling pathway [17,18]. In this study, we reported the upregulation of miR-1269b in IHs. Moreover, miR-1269b also increased the proliferation of hemangioma-derived HDECs. Therefore, miR-1269b may promote IHs by stimulating cell proliferation.

The present study reported the direct interaction between BRCAT54 and miR-1269b in HDECs, but not in HUVECs, and detected BRCAT54 in both cytoplasm and nuclear of both HDECs and HUVECs. In addition, BRCAT54 suppressed the role of miR-1269b in promoting the proliferation of HUVECs, while these two ncRNAs showed no role in mediating the proliferation of HUVECs. Therefore, BRCAT54 may sponge miR-1269b only in hemangioma-derived HDECs, but not in normal HUVECs, to regulate IHs. Therefore, the overexpression of BRCAT54 may serve as a target to suppress cell proliferation in IHs. However, novel techniques are needed to deliver BRCAT54 into vascular tissues.

Interestingly, BRCAT54 is an inhibitor of cell proliferation, but its accumulation was increased during the proliferation stage. In contrast, the accumulation of miR-1296b, which was an activator of cell proliferation, was decreased in proliferation stage. Studies have shown that cell proliferation inhibitors are usually downregulated in IHs [14], while cell proliferation activators are usually upregulated in IHs [15]. It is speculated that there are multiple inhibitors and activators in IHs, and they could interact to regulate the expression of each other. Our findings reveal the complexity of the molecular mechanism of IHs.

## Conclusion

BRCAT54 is downregulated in IHs, and miR-1269b is highly upregulated in IHs. BRCAT54 may sponge miR-1269b to regulate the proliferation of HDECs, thereby participating in IHs. The exogenous application of BRCAT54 may serve as a potential target to treat IHs. However, novel RNA delivery techniques are needed to efficient deliver BRCAT54 to target sites.

## Data Availability

The data that support the findings of this study are available from the corresponding author upon reasonable request.

## Highlights

1. Involuting-phase tissues exhibited decreased expression levels of BRCAT54 and increased expression levels of miR-1269b;
2. BRCAT54 was detected in both nuclear and cytoplasmic fractions, and it interacted with miR-1269b;
3. BRCAT54 suppressed the role of miR-1269b in enhancing the proliferation of HDECs.
